# Enhanced phenolic compounds tolerance response of *Clostridium beijerinckii* NCIMB 8052 by inactivation of Cbei_3304

**DOI:** 10.1186/s12934-018-0884-0

**Published:** 2018-03-03

**Authors:** Jun Liu, Qinlu Lin, Xueying Chai, Yunchuan Luo, Ting Guo

**Affiliations:** 1 National Engineering Laboratory of Rice and By-Product Deep Processing, Central South University of Forestry and Technology, Shaoshan Nan Road No. 498, Changsha, 410004 People’s Republic of China; 2 College of Food Science and Technology, Central South University of Forestry and Technology, Shaoshan Nan Road No. 498, Changsha, 410004 People’s Republic of China

**Keywords:** *C. beijerinckii*, Phenolic compound, Butanol, Transmembrane, Transcriptome analysis

## Abstract

**Background:**

Phenolic compounds generated in hydrolysis of lignocellulosic materials are major limiting factors for biological production of solvents by *Clostridia*, but it lacks the attention on the study of adaptation or resistance mechanisms in response to phenolic compounds.

**Results:**

Gene Cbei_3304, encoding a hypothetical membrane transport protein, was analyzed by bioinformatic method. After insertional inactivation of the functionally uncertain gene Cbei_3304 in *Clostridium beijerinckii* NCIMB 8052, resulted in enhanced phenolic compounds tolerance. Compared to the parent strain *C. beijerinckii* NCIMB 8052, evaluation of toxicity showed the recombination stain *C. beijerinckii* 3304::int had a higher level of tolerance to four model phenolic compounds of lignocellulose-derived microbial inhibitory compounds. A comparative transcriptome analysis showed that the genes were involved in membrane transport proteins (ABC and MFS family) and were up-regulated expression after disrupting gene Cbei_3304. Additionally, the adaptation of *C. beijerinckii* NCIMB 8052 in response to non-detoxified hemicellulosic hydrolysate was improved by disrupting gene Cbei_3304.

**Conclusion:**

Toxicity evaluation of lignocellulose-derived phenolic compounds shows that Cbei_3304 plays a significant role in regulating toxicities tolerance for ABE fermentation by *C. beijerinckii*, and the adaptation of non-detoxified hemicellulosic hydrolysate is significantly improved after inactivation of Cbei_3304 in wild-type strain *C. beijerinckii* NCIMB 8052. It provided a potential strategy for generating high inhibitor tolerance strains for using lignocellulosic materials to produce solvents by *clostridia* in this study.

**Electronic supplementary material:**

The online version of this article (10.1186/s12934-018-0884-0) contains supplementary material, which is available to authorized users.

## Background

Compared to ethanol, butanol plays an important role in the overall success of the biofuels industry, because its qualities are more similar to those of gasoline [1]. Currently, the use of renewable lignocellulosic materials such as corn fiber or cob, wheat straw, bagasse fiber as a substrate for butanol production has been widely investigated to reduce the fermentation cost. However, a range of lignocellulose-derived microbial inhibitory compounds (LDMICs) are generated along with sugars during pretreatment [2], and significantly inhibit the cell growth and metabolism by penetrating into biological membranes increasing cell fluidity, diminishing proton motive force, reducing ATP levels, causing DNA mutagenesis and inhibiting essential enzymes [3, 4]. Especially, phenolic compounds in the *Clostridium beijerinckii*-induced ABE (acetone–butanol–ethanol) fermentation response, dramatically inhibit the cell growth and solvent production [5]. In addition, due to the diversity of these phenolic compounds, which have various functional groups such as aldehyde, ketone, acid and alcohol, and the side groups such as methoxy and hydroxyl groups [6], the antimicrobial activity is different and the exact mechanism of toxicity to *C. beijerinckii* is still uncertain.

Generally, the toxicity resistance pathways usually focus on the process of metabolizing them to less toxic compounds, reducing the toxicity concentration in the cytoplasm by limiting the uptake or enhancing the extrusion [7], and the regulation factors involved in the toxicity tolerance such as heat shock protein and transcription factors. The group of toxic compounds metabolism: Furfural and 5-hydroxymethyl-furfural (HMF) are converted into alcohol dependent on the intracellular energy and reducing power (NADH/NADPH) to reduce the toxicity [4, 8, 9]; Phenolic compounds such as ρ-coumaric acid, ferulic acid, and caffeic acid are metabolized by phenolic acid decarboxylase and reductase in *Lactobacillus* spp. and *Saccharomyces cerevisiae* [10, 11]; Our previous research reported that the gene Cbei_4693, probably encoded a NADPH-dependent FMN reductase, plays an important role in regulating ferulic acid tolerance of the ABE fermentation by *C. beijerinckii*, and ferulic acid could be completely converted into the less toxic phenolic compound–hydroferulic acid [12]. The group of regulation factors involved in the tolerance of toxic compounds: Overexpression of *groES* and *groEL* genes, encoding head shock proteins, dramatically improved the production of acetone and butanol even under 0.5 g/L of ferulic acid stressed condition [13]; The Multiple antibiotic resistance Regulator (MarR) family, a transcription factor such as *hosA* protein, is associated with the regulation of genes that are involved in antibiotic resistance and detoxification noxious compounds to *Enterobacteriaceae* spp. [14]; In addition, there are two major types of efflux pump involved in microorganism resistance of toxicities: ATP-binding cassette (ABC) transporters and major facilitator superfamily (MFS) transporters. *MDR1*, a MFS transporter, was identified by its ability to confer benomyl and methotrexate resistance on *S. cerevisiae*. Becker et al. [15] have reported that disrupting the gene *CaMDR1* markedly reduced virulence of *Candida albicans* in an animal model. To survive in toxic inorganic compounds (such as arsenite) environment, organisms have developed the resistance pathways such as arsenic exclusion through various membrane-bound transporters [16]. However, there is a lack of attention to study adaptation or resistance mechanisms of phenolic compounds in the *C. beijerinckii* fermentation.

Generally, the cell growth, substrate utilization, ABE production and the toxic compounds tolerance are significantly improved in calcium carbonate-mediated fermentation medium by *C. beijerinckii* NCIMB 8052 in non-detoxified hemicellulosic hydrolysate. Proteomic and biochemical analysis were used to elucidate the role of calcium in ABE fermentation, a protein (YP_001310387.1, Hypothetical protein) was 11.5-fold down-regulated in the CaCO_3_-supplemented cultures relative to the control [17]. Use of the bio-informatic analysis and the phylogenetic tree of proteins to determine the function of hypothetical protein, and the results showed that the protein YP_001310387.1 was encoded by gene Cbei_3304 and speculated to be a membrane transporter in *C. beijerinckii* NCIMB 8052. In this study, we speculated that Cbei_3304 plays an important role in lignocellulose-derived microbial inhibitory compounds resistance, especially the phenolic compounds. Then, inactivation of Cbei_3304 to evaluate the phenolic compounds tolerance and the adaptation of non-detoxified hemicellulosic hydrolysate treated with dilute sulfuric acid in ABE fermentation by *C. beijerinckii* NCIMB 8052 was studied. In addition, a comparative transcriptome analysis of wild-type and recombination strain was carried out to further illuminate the effect of Cbei_3304 on transport system in *C. beijerinckii* NCIMB 8052.

## Results and discussion

### Bioinformation analysis of gene Cbei_3304

**Fig. 1:**
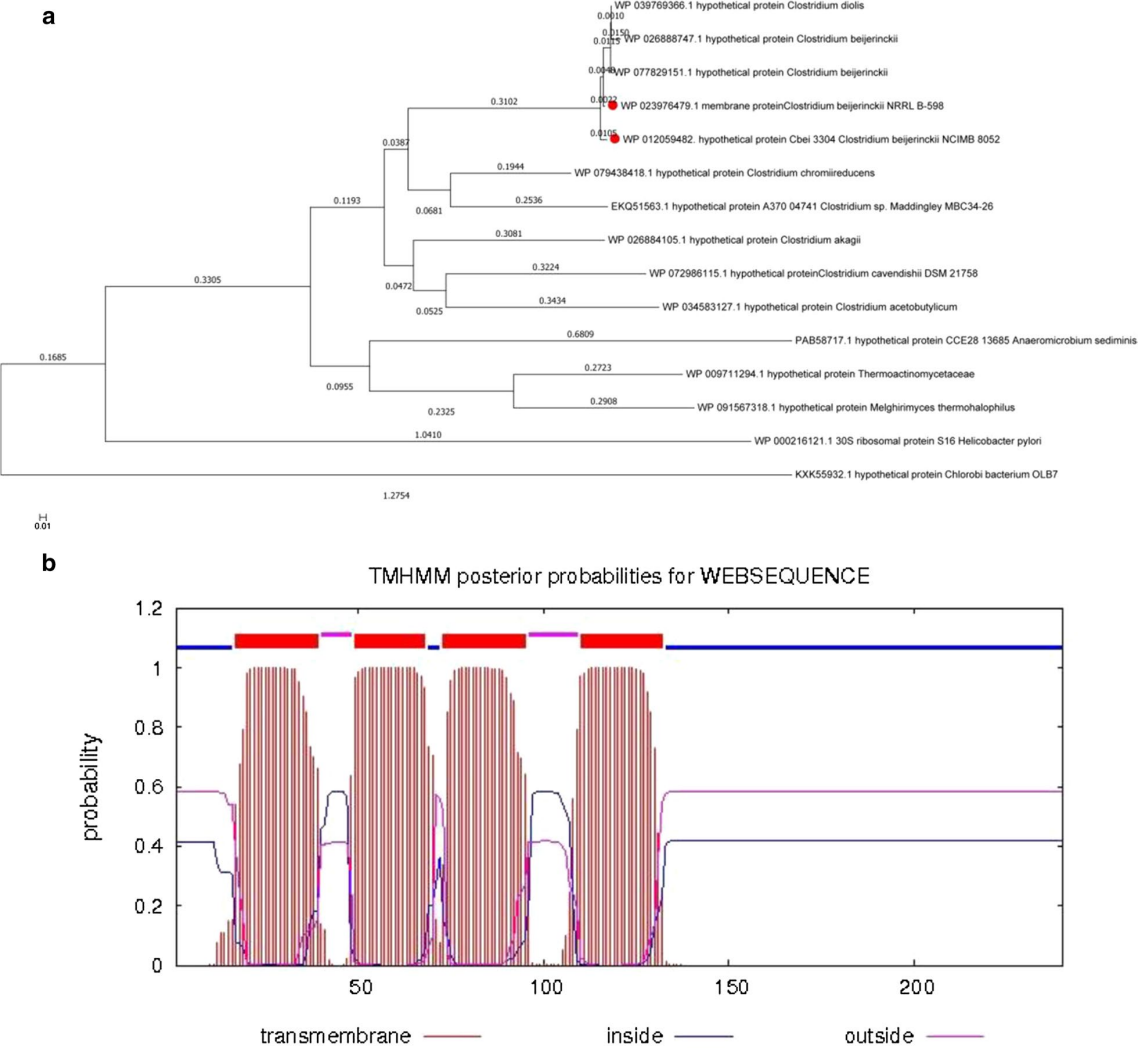
Bioinformation analysis of gene Cbei_3304. Neighbor-joining trees of proteins using MEGA6.0 analysis (**a**), the function and structure analysis of gene Cbei_3304 by TMHMM Server v. 2.0 (**b**)

The protein YP_001310387.1, which was dramatically down-regulated in calcium carbonate-mediated fermentation medium by *C. beijerinckii* NCIMB 8052 in response to ferulic acid, was identified as the hypothetical protein Cbei_3304 (the sequences of gene Cbei_3304 and protein were showed in Additional file 1: Figure S1) by searching the NCBI protein database (https://www.ncbi.nlm.nih.gov/protein/YP_001310387.1?report=genpept). To confirm the function of this hypothetical protein, neighbor-joining trees of proteins (Molecular Evolutionary Genetics Analysis Version 6.0) and CBS Prediction Servers-Protein function and structure (TMHMM Server v. 2.0, http://www.cbs.dtu.dk/services/TMHMM-2.0/) were applied. In the phylogenetic tree, although they were divided into at least two sub-trees and still heavily focused on hypothetical protein in different *Clostridia* strains, the hypothetical protein Cbei_3304 was mostly homologous to the membrane protein (100% Query Cover, 99% Identity) from *C. beijerinckii* NRRLB-598 (Fig. 1a). To further verify the above result of membrane protein, the function of gene Cbei_3304 was analyzed by CBS Prediction Servers-Protein function and structure. Figure 1b showed that the total number of the length of protein Cbei_3304 sequence is 240 aa and there are 4 predicted transmembrane helices (the position is 17aa–39aa, 49aa–68aa, 73aa–95aa and 110aa–132aa, respectively). In addition, the expected number of amino acids in-transmembrane helices is 87.97623, which is significantly larger than 18. According to bioinformation analysis, we speculated that Cbei_3304 protein is very likely to be a transmembrane protein.

### Effect of gene Cbei_3304 on phenolic compounds tolerance

**Fig. 2:**
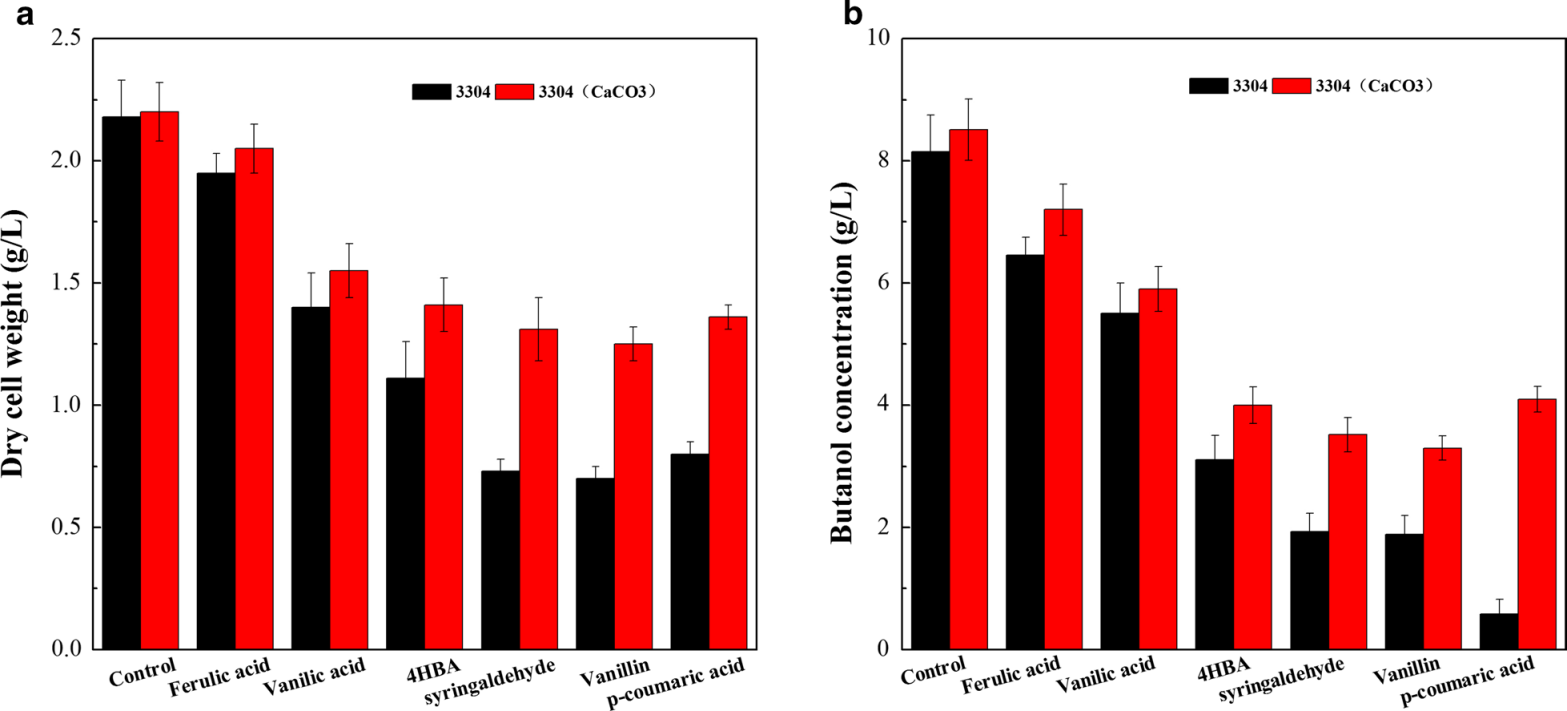
Cells growth (**a**) and butanol production (**b**) of *C. beijerinckii* 3304::int. Cells were grown in screw-capped bottles in fermentation medium containing 30 g/L glucose and 0.5 g/L of six model phenolic compounds. 3304: strain *C. beijerinckii* 3304::int; 3304(Ca): *C. beijerinckii* 3304::int with 3 g/L of CaCO_3_ supplemented

To investigate the effect of Cbei_3304 protein on cell growth of *C. beijerinckii* NCIMB 8052 and butanol production in phenolic compounds-supplemented P2 medium. Batch-fermentations with different phenolic compounds (the concentration was 0.5 g/L, respectively) were carried out by wild-type strain *C. beijerinckii* NCIMB 8052 and recombination strain *C. beijerinckii* 3304::int for 96 h. Compared to wild-type stain *C. beijerinckii* NCIMB 8052 (data were shown in our previous paper [18]), disrupting Cbei_3304 significantly enhanced butanol production and cell growth in response to the phenolic compounds, especially ferulic acid and vanilic acid. 1.90 and 1.38 g/L of DCW (Fig. 2a), 6.45 and 5.5 g/L of butanol (Fig. 2b) were produced in P2 medium with 0.5 g/L of ferulic acid and 0.5 g/L of vanilic acid by *C. beijerinckii* 3304::int, respectively. However, *C. beijerinckii* 3304::int did not exhibit high tolerance of other phenolic compounds, especially ρ-coumaric acid, and the exact cause was still unclear. Besides, the tolerance of the complementation mutant strain *C. beijerinckii* 3304::cp to the six model phenolic compounds (0.5 g/L) was almost same as that by the recombination stain *C. beijerinckii* 3304::int (data not showed), it indicated that Cbei_3304 in the expression plasmid pWD1-3304 was incompletely expressed or loss of expression after transforming into *C. beijerinckii* 3304::int. Based on the antimicrobial activity of phenolic compounds is determined by their chemical structure, then we speculated that the ability of penetration into intracellular or cytomembrane of these compounds against *C. beijerinckii* was different by their different chemical structures, and the suspected transmembrane protein Cbei_3304 did not relate to regulate ρ-coumaric acid tolerance. Then, the low butanol production and cell growth of *C. beijerinckii* 3304::int were almost same as that by wild-type strain in P2 medium with 0.5 g/L of ρ-coumaric acid-supplemented.

Given that the gene Cbei_3304 was significantly down-regulated 11.5-fold in the CaCO_3_-supplemented cultures relative to the control to illustrate the effect of calcium ion involved in regulation of the butanol tolerance and cell growth [17]. It suggests that both calcium ion and Cbei_3304 play an important role in regulating phenolic compounds tolerance, but the exact role of them to different kinds of phenolic compounds was still uncertain. Then, batch fermentations were carried out with different kinds of phenolic compounds (0.5 g/L) and 3.0 g/L CaCO_3_ by *C. beijerinckii* 3304::int. Interestingly, although CaCO_3_ supplementation indeed improved the production of butanol and the cell growth by *C. beijerinckii* 3304::int in fermentation medium with different phenolic compounds, especially ρ-coumaric acid, the concentration of butanol was dramatically enhanced to 4.1 from 0.58 g/L under the condition of P2 medium without CaCO_3_ supplementation, and the value of DCW of *C. beijerinckii* 3304::int was almost improved for two fold. However, the butanol production and cell growth of *C. beijerinckii* 3304::int in ferulic acid, vanilic acid and 4HBA-supplemented medium were almost the same as that in the condition of CaCO_3_ supplementation. On the basis of the above results, when the concentration of phenolic compounds at 0.5 g/L in the medium, calcium ion just dramatically involves in enhancing tolerance of some phenolic compounds (such as ρ-coumaric acid, vanillin and syringaldehyde) but not all by recombination strain *C. beijerinckii* 3304::int, especially ferulic acid, and inactivation of gene Cbei_3304 in wild-type strain *C. beijerinckii* NCIMB 8052 obtained an adequate capacity of enhanced ferulic acid tolerance (Additional file 3: Table S2).

### DEGs by inactivation of Cbei_3304

**Fig. 3:**
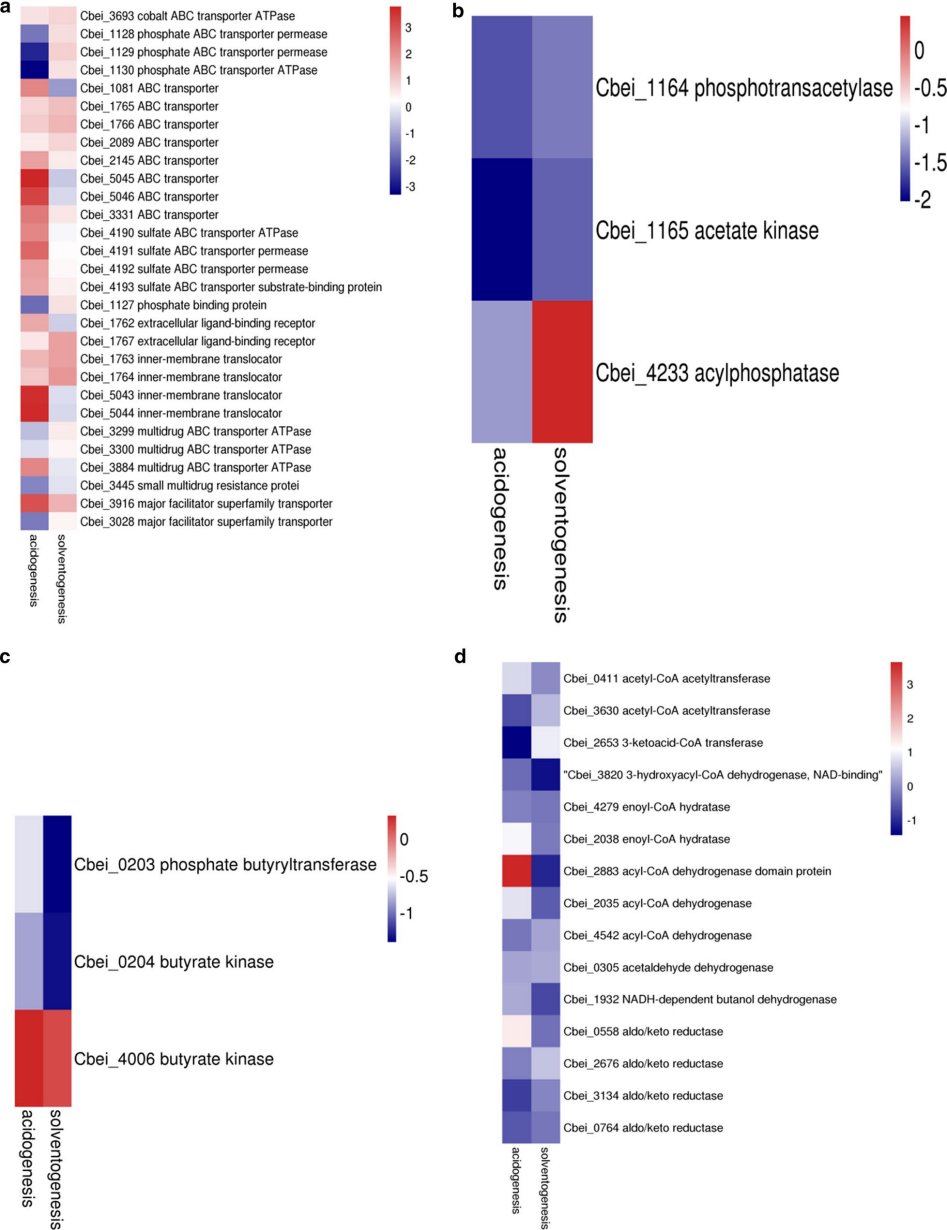
Comparison of gene expression after inactivation of gene Cbei_3304 against *C. beijerinckii* NCIMB 8052 at the acidogenic and solventogenic phases. Results were grouped into different attributes: membrane transport genes (**a**); acetate formation gene (**b**); butyrate formation genes (**c**); butanol formation genes (**d**)

To clarify the exact effect of Cbei_3304 on the growth and fermentation metabolism of *C. beijerinckii* NCIMB 8052, a comparative transcriptome analysis was studied by RNA-seq. Batch fermentations of *C. beijerinckii* NCIMB 8052 and 3304::int were carried out in 250-mL screw-capped bottles containing 100 mL P2 medium, respectively, after inoculation (10% v/v) for 12 h as the acidogenesis and 36 h as the solventogenesis. Given that gene Cbei_3304 suspected to be a transmembrane protein, DEGs involved in ATP-binding cassette (ABC) transporters and major facilitator superfamily (MFS) transporters as well as butanoate metabolism were highly represented in the comparative transcriptome analysis. The genes involved in membrane transport proteins (ABC and MFS family) almost were up-regulated expressions after disrupting gene Cbei_3304 in wild-type strain (Fig. 3a). In acidogenic phase, genes encoding ABC transporter (Cbei_2145, Cbei_3331, Cbei_5045 and Cbei_5046) and sulfate ABC transporter permease (Cbei_4190–Cbei_4193) were more highly expressed, especially the gene Cbei_5045 and Cbei_5046 was upregulated by more than 13- and 9-fold, respectively; In addition, the gene Cbei_5043 and Cbei_5044, encoding inner-membrane translocator, were both dramatically up-regulated by more than 12-fold; However, the phosphate ABC transporter (Cbei_1127–Cbei_1130), catalyzing the chemical reaction ATP + H_2_O + phosphate_(out)_ = ADP + phosphate + phosphate_(in)_ (https://en.wikipedia.org/wiki/Phosphate-transporting_ATPase), were significantly down-regulated, which indicated that the transportation of ferulic acid and vanilic acid across cell membrane very likely does not relate to energy, such as ATP. Inexplicably, compared to the wild-type strain, the genes involved in encoding transmembrane proteins were almost unchanged in solventogenic phase by the recombination strain *C. beijerinckii* 3304::int. The results illustrated that the role of gene Cbei_3304 relates to the cell transport system mainly in acidogenesis rather than in solventogenesis (Additional file 2: Table S1).

However, the ABE fermentation metabolism containing acetate (Fig. 3b, Additional file 3: Table S2) and butyrate formation (Fig. 3c, Additional file 4: Table S3) and butanol metabolism (Fig. 3d, Additional file 5: Table S4) was weakly down-regulated after inactivation of gene Cbei_3304. The expression of aldo/keto reductase (Cbei_0558, Cbei_0764, Cbei_2676 and Cbei_3134) was down-regulated by one time, but the NADH-dependent butanol dehydrogenase (*bdh*) was significantly down-regulated by more than 1.6-fold in solventogenic phase. In addition, the genes involve in the production of acetate and butyrate were also weakly down-regulated throughout the whole fermentation period, mainly focused on the gene Cbei_0203 and Cbei_0204, and Cbei_1164 and Cbei_1165, respectively.

In addition, a comparative transcriptome analysis of *C. beijerinckii* NCIMB 8052 and 3304::int on adaptation mechanisms in response to ferulic acid or vanilic acid (ferulic acid or vanilic acid supplemented into P2 medium) would be further investigated to clarify the mechanism of enhanced phenolic acid tolerance in detail.

### Evaluation of butanol production using non-detoxified hemicellulosic hydrolysate

The concentrations of total reducing sugar and inhibitors about raw hemicellulosic hydrolysate treated with dilute sulfuric acid (SAHHC and SAHHB) after sterilization were showed in Tables 1 and 2, respectively. The raw SAHHC containing 60 g/L of reducing sugar (60P2-SAHHC) was diluted to desired sugar concentrations as follows: 30P2-SAHHC (containing 30 g/L of reducing sugar), 40P2-SAHHC (containing 40 g/L of reducing sugar). After sterilizing, TPC concentrations of 60P2-SAHHC, 40P2-SAHHC, and 30P2-SAHHC were 4.72, 2.71 and 2.23 g/L, respectively. Analogously, the non-detoxified SAHHB was diluted to desired sugar concentrations as 46P2-SAHHB, 40P2-SAHHB, and 30P2-SAHHB, and the concentration of TPC was 3.57, 2.39 and 1.87 g/L after sterilizing, respectively. Additionally, although the concentrations of the six model phenolic compounds were both significantly low in SAHHC and SAHHB, the combination of these inhibitors present synergistic toxicity effects, which can dramatically enhance the inhibition ability on cell growth and butanol production by *C. beijerinckii* [19].

**Table 1:** The concentration of total reducing sugars, TPC and phenolic compounds of SAHHC after sterilization at 115 °C for 20 min

	60P2-SAHHC	40P2-SAHHC	30P2-SAHHC
Total sugars	60.21 ± 2.12 g/L	38.24 ± 2.11 g/L	28.23 ± 2.23 g/L
TPC	4.72 ± 0.11 g/L	2.71 ± 0.08 g/L	2.23 ± 0.09 g/L
Ferulic acid	50.04 ± 0.07 mg/L	36.36 ± 0.03 mg/L	19.89 ± 0.07 mg/L
Vanilic acid	37.86 ± 0.04 mg/L	26.67 ± 0.05 mg/L	20.05 ± 0.05 mg/L
Vanilin	20.83 ± 0.05 mg/L	14.01 ± 0.08 mg/L	10.06 ± 0.04 mg/L
ρ-Coumaric acid	48.41 ± 0.03 mg/L	32.67 ± 0.07 mg/L	25.01 ± 0.06 mg/L
4-HBA	45.58 ± 0.08 mg/L	33.33 ± 0.04 mg/L	23.11 ± 0.07 mg/L
Syrangaldehyde	62.82 ± 0.14 mg/L	42.31 ± 0.06 mg/L	32.04 ± 0.09 mg/L
HMF	510 ± 20 mg/L	351 ± 14 mg/L	260 ± 21 mg/L
Furfural	810 ± 40 mg/L	550 ± 17 mg/L	407 ± 25 mg/L

**Table 2:** The concentration of total reducing sugars, TPC and phenolic compounds of SAHHB after sterilization at 115 °C for 20 min

	46P2-SAHHB	40P2-SAHHB	30P2-SAHHB
Total sugars	43.21 ± 2.08 g/L	39.12 ± 1.13 g/L	27.23 ± 2.14 g/L
TPC	3.57 ± 0.15 g/L	2.39 ± 0.07 g/L	1.87 ± 0.11 g/L
Ferulic acid	35.03 ± 0.09 mg/L	23.26 ± 0.05 mg/L	16.99 ± 0.04 mg/L
Vanilic acid	39.06 ± 0.05 mg/L	26.17 ± 0.08 mg/L	18.15 ± 0.09 mg/L
Vanilin	26.73 ± 0.07 mg/L	17.31 ± 0.07 mg/L	14.06 ± 0.08 mg/L
ρ-Coumaric acid	50.41 ± 0.03 mg/L	34.17 ± 0.04 mg/L	27.02 ± 0.10 mg/L
4-HBA	35.18 ± 0.08 mg/L	23.33 ± 0.04 mg/L	17.11 ± 0.07 mg/L
Syrangaldehyde	72.82 ± 0.14 mg/L	48.71 ± 0.12 mg/L	35.04 ± 0.11 mg/L
HMF	470 ± 35 mg/L	320 ± 30 mg/L	240 ± 26 mg/L
Furfural	766 ± 50 mg/L	516 ± 28 mg/L	390 ± 24 mg/L

**Fig. 4:**
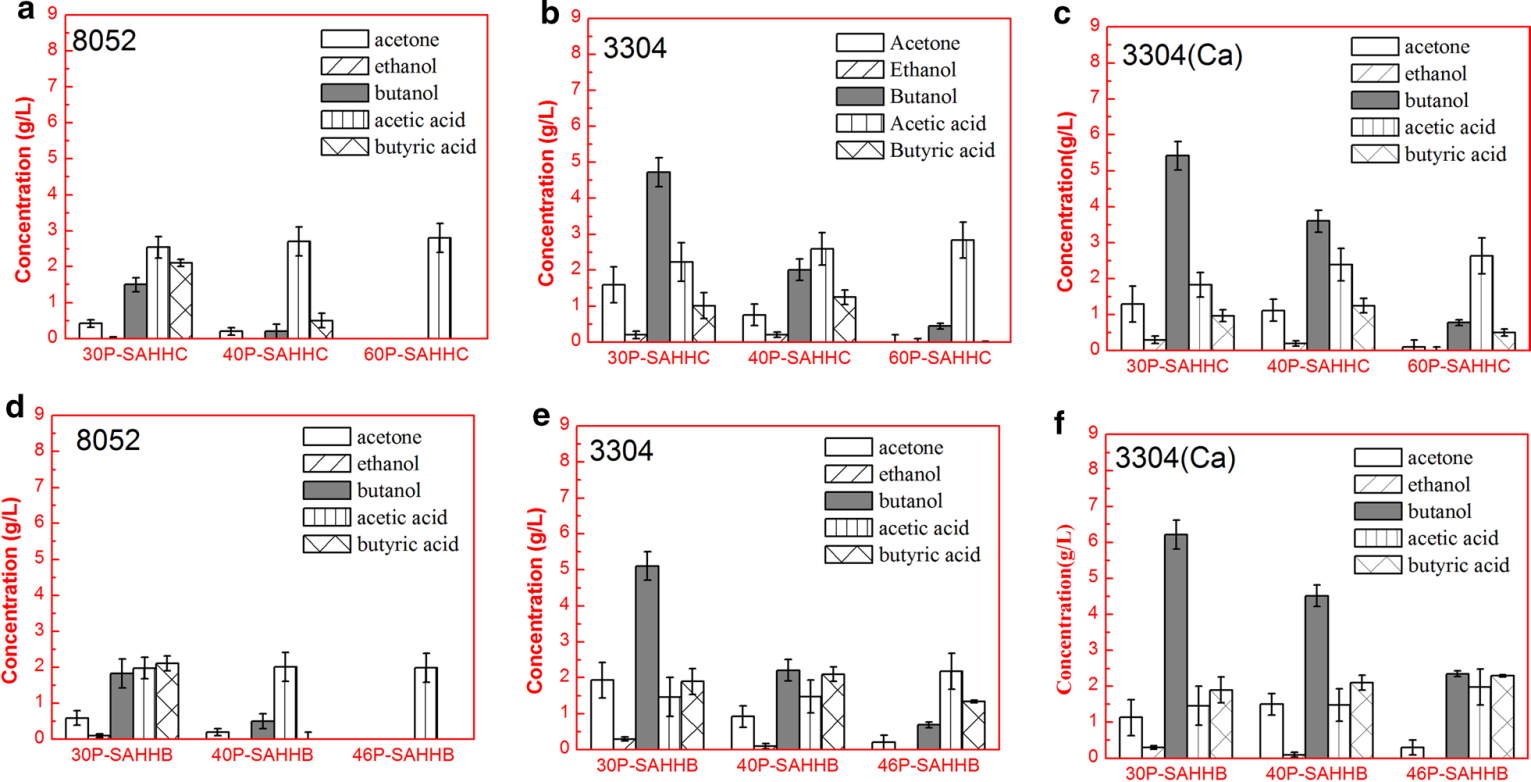
ABE bottle batch fermentations using non-detoxified hemicellulosic hydrolysate of corn cob treated with dilute sulfuric acid (SAHHC) and bagasse fiber treated with dilute sulfuric acid (SAHHB) containing different concentrations of total reducing sugars by *C. beijerinckii* NCIMB 8052 and 3304::int for 96 h. *C. beijerinckii* NCIMB 8052 in SAHHC (**a**); *C. beijerinckii* 3304::int in SAHHC (**b**); *C. beijerinckii* 3304::int in SAHHC with 3 g/L of CaCO_3_ supplemented (**c**); *C. beijerinckii* NCIMB 8052 in SAHHB (**d**); *C. beijerinckii* 3304::int in SAHHB (**e**); *C. beijerinckii* 3304::int in SAHHB with 3 g/L of CaCO_3_ supplemented (**f**)

The recombination stain *C. beijerinckii* 3304::int has showed high phenolic compounds tolerance, thus we further investigated the adaptation and fermentation ability in different non-detoxified SAHHC and SAHHB. 6.52 g/L (4.72 g/L of butanol, 1.59 g/L of acetone, and 0.21 g/L of ethanol; showed in Fig. 4b) and 7.34 g/L (5.11 g/L of butanol, 1.93 g/L of acetone, and 0.30 g/L of ethanol; showed in Fig. 4e) of total solvents was produced by *C. beijerinckii* 3304::int using 30P-SAHHC (2.23 g/L of TPC) and 30P-SAHHB (1.87 g/L of TPC), respectively, and CaCO_3_ supplementation did not significantly increase the production of butanol (Fig. 4c, f), suggesting that high phenolic compounds tolerance of *C. beijerinckii* 3304::int and the inhibitor concentration were at a lower level. In addition, in 40P-SAHHC (2.71 g/L of TPC) and 40P-SAHHB (2.39 g/L of TPC) basic medium, *C. beijerinckii* 3304::int just produced 2.97 g/L (2.01 g/L of butanol, 0.76 g/L of acetone, and 0.20 g/L of ethanol; showed in Fig. 4b) and 3.23 g/L (2.21 g/L of butanol, 0.92 g/L of acetone, and 0.10 g/L of ethanol; showed in Fig. 4e) of total solvents. The total concentration of ABE produced using SAHHB was higher than that using SAHHC by *C. beijerinckii* 3304::int, because of the much higher concentration of TPC or inhibitors in SAHHC. Disappointingly, because of a quite high concentration of inhibitors, there was almost no butanol production using raw and non-detoxified SAHHC (60P) and SAHHB (46P) by *C. beijerinckii* NCIMB 8052 after disrupting the suspected transmembrane protein, and even though 3.0 g/L of CaCO_3_ was supplemented to enhance the inhibitors tolerance and adaptation ability, the production of butanol was still at a low level. Fortunately, compared to the wild-type strain, the adaptation and tolerance of non-detoxified hemicellulosic hydrolysate for ABE fermentation to butanol production were significantly enhanced by inactivation of Cbei_3304, and the cost of industrial-scale production could be decreased by using this novel approach to generate high inhibitor tolerance strains rather than CaCO_3_ supplementation is used to detoxify the hemicellulosic hydrolysate.

## Conclusions

The gene Cbei_3304 was found and analyzed to be a suspected membrane transport protein by two kinds of bio-informatic methods in this study. Evaluation of lignocellulose-derived phenolic compounds toxicity showed gene Cbei_3304 played a significant role in regulating toxicities tolerance for ABE fermentation by *C. beijerinckii*, and the adaptation of non-detoxified hemicellulosic hydrolysate was significantly improved after inactivation of gene Cbei_3304 in wild type strain *C. beijerinckii* NCIMB 8052. The results provided a novel approach for generating high inhibitor tolerance strains for using lignocellulosic materials to produce solvents by *clostridia*.

## Methods

### Chemicals and materials

Ferulic acid, vanilic acid, vanilin, ρ-coumaric acid, syrangaldehyde, 4-hydroxybenzoic acid (4HBA), Furfural, 5-hydroxymethyl furfural (HMF), tannin and tetracycline, ampicillin, erythromycin were purchased from Sigma Chemicals. Yeast extract and peptone were obtained from Oxoid Ltd (Thermo Fisher Biochemical, Beijing, China). Other chemicals and laboratory media of analytical grade were purchased from Sangon Biological Engineering Technology and Services Co. Ltd (Shanghai, China).

The corn fiber (passed through 30–40 mesh screens) was purchased from Shandong Yanggu Shengda corn cob granule Co. Ltd (Shandong, China). The bagasse fiber was donated from Guangzhou Sugarcane Industry Research Institute (Guangdong, China).

### Bacterial strains and culture medium

*Clostridium beijerinckii* cells were inoculated in YPS medium (3.0 g yeast extract, 5.0 g peptone, 10.0 g soluble starch, 2.0 g ammonium acetate, 2.0 g NaCl, 3.0 g MgSO_4_·7H_2_O, 1.0 g KH_2_PO_4_, 1.0 g K_2_HPO_4_, 0.1 g FeSO_4_·7H_2_O per liter; solid medium with 20 g/L agar powder) with addition of 10 µg/mL erythromycin as required at 37 °C, anaerobically. Cells were sub-cultured in fresh YPS medium with a 5% inoculum size for 8 h to reach an optical density at 600 nm (OD_600_) of approximately 2.0 (secondary seed cells) for ABE fermentation. *Escherichia coli* DH5α and TOP 10 cells were cultured in Luria–Bertani (LB) broth (0.5 g yeast extract, 1.0 g peptone, 1.0 g NaCl in 100 mL distilled water; Solid medium with 20 g/L agar powder) with 15 µg/mL tetracycline and 50 µg/mL ampicillin as required.

### Construction of recombination strain

The bacterial strains, plasmids and primers used were listed in Table 3.

**Table 3:** Bacterial strains, plasmids and primers used in this study

Strain/plasmid/primer	Relevant characteristics	Reference
Strains		
NCIMB 8052	Wild-type	NCIMB
3304::int	Group II intron inserted at 101/102a of Cbei_3304	This study
3304::cp	3304::int harboring 3304-expression vector	This study
3304::int-pWD1	3304::int harboring control vector pWD1	This study
*E. coli* DH5α	Cloning	Invitrogen
*E. coli* Top10	Harboring pAN2 plasmid	Invitrogen
Plasmids		
pAN2	Methylation plasmid, *Φ3T I, p15A ori, Tet*^*R*^	[20]
pWJ1	Derived from pSY6 with pCB102 ORI instead of pIM13 ORI	[21]
pWJ1-3304	Derived from pWJ1 for intron insertion in Cbei_3304 at 101/102a	This study
pWD1-3304	Derived from pWJ1, with Cbei_3304 expressing cassette added	This study
Primers	Sequence (5′→3′)	
pWJ1-101-F	GGAGTGTCGAGGATC*CTCGAG*ATTATCCTTATAATTCAAAGC	This study
pWJ1-101-R	GGTTCTCCTACAGAT*TGTACA*AGTCAAAGCACATAACTTAC	This study
pWD1-101-F	GGAGTGTCGAGGATC*CTCGAG*ATGACAAAGGTTAATAAATT	This study
pWD1-101-R	CAGATTGTACTGAGAGTGCAC*CATATG*TAATCAACATTGATGGAAT	This study
3304-Test-F	ATGCGGATCCATGACAAAGGTTAATAAATT	This study
3304-Test-R	GTACGAATTCTTAATCAACATTGATGGAAT	This study

Construction of the inactivation plasmid pWJ1-3304 was performed as follows: 101/102a position for gene Cbei_3304 insertion was chosen using the Clostron system and group II intron fragments was generated (http://www.clostron.com); Subsequently, the group II intron fragments was synthesized using the primers pWJ1-101-F and pWJ1-101-R and constructed plasmids pWJ1-3304 using the infusion one-step clone kit (Vazyme Biotech Inc., Nanjing, China) by inserting group II intron fragments into *Xho*I and *Bsr*GI restriction sites of the pWJ1 plasmid.

Construction of the expression plasmid pWD1-3304 was performed as follows: the full length CDS of gene Cbei_3304 was amplified using primers pWD1-101-F and pWD1-101-R and the *C. beijerinckii* NCIMB 8052 genome DNA as a template. The carrier vector was digested by the *Xho*I and *Nde*I restriction enzyme. Followed, gene Cbei_3304 fragments were purified and cloned into pWJ1 using the infusion one-step clone kit.

Plasmids pWJ1-3304 and pWD1-3304 were initially methylated in *E. coli* TOP10 (pAN2), then transformed via electroporation and colony PCR by the primers 3304-Test-F and 3304-Test-R was used for screening and isolating inactivated mutants (*C. beijerinckii* 3304::int) and complementation mutants (*C. beijerinckii* 3304::cp) [22].

### Pre-treatment and hydrolysis of lignocellulosic materials

100 g of lignocellulosic materials (corn cob or bagasse fiber) was soaked in 500 mL of dilute (2% w/v) sulphuric acid in a 1 L triangular flask, then was hydrolysed in an autoclave at 125 °C for 150 min. Raw hydrolysate was neutralized to pH 6.6 with solid Ca(OH)_2_ at 50 °C and was filtered through filter paper to remove the solid materials. The liquid filtrate (SAHHC and SAHHB) was collected for total sugar and soluble phenolic compounds (TPC, six model phenolic compounds and furfurans) detection and was used as the carbon source for batch-fermentation studies [23].

### Screwed-bottle batch fermentation

Batch fermentation was carried out in 100 mL screw-capped bottles containing 45 mL fermentation medium (P2 medium or SAHHC and SAHHB as a carbon source) and 5 mL secondary seed cells (volume inoculum size 10%) without agitation or pH control in an anaerobic condition at 37 °C. P2 medium contains P2 stock solutions (buffer solution, mineral solution, and vitamin solution) and carbon source with addition of 10 µg/mL erythromycin as required (fermentation by *C. beijerinckii* 3304::cp), followed by sterilization at 115 °C for 20 min [24].

The fermentation medium (P2 medium) contained the following components: carbon source (30 g/L glucose); phosphate buffer (0.5 g/L KH_2_PO_4_ and 0.5 g/L K_2_HPO_4_), ammonium acetate (2.2 g/L); vitamin solution (1 mg/L ρ-amino-benzoic acid, 1 mg/L thiamine, and 0.01 mg/L biotin); and mineral solution (0.01 g/L MnSO_4_·H_2_O, 0.01 g/L NaCl, 0.2 g/L MgSO_4_·7H_2_O, and 0.01 g/L FeSO_4_·7H_2_O).

After fermentation, 2 mL fermentation supernatant was collected to detect the concentration of butanol; 2 mL fermentation culture was collected for evaluation of dry cell weight.

### Analytical methods

Butanol concentration was analyzed using a gas chromatograph (7890A, Agilent, Wilmington, DE, USA) equipped with a flame ionization detector and an Agilent HP-INNOWAX column (0.25 mm × 60 m). The oven was programmed to heat from 70 to 190 °C at a rate of 20 °C/min, with an initial holding time of 0.5 min and a post-holding time of 4 min. The injector and detector temperatures were programmed to 180 and 220 °C, respectively. Nitrogen was used as the carrier gas at a flow rate of 30 mL/min [25].

Total sugar concentration was measured according to the 3,5-dinitrosalicyclic acid (DNSA) method. Dry cell weight (DCW) was computed from a curve of OD_600_ versus dry weight; an OD_600_ of 1.0 represented 260 mg dry weight. Total phenolic compounds concentration (TPC) was determined by the Folin–Ciocalteu method using vanillin and tannin as standards [26]. The concentration of model phenolic compounds and furans was determined by high-performance liquid chromatography analysis (Agilent 1200 series; Hewlett-Packard, Wilmington, DE, USA) at 280 nm; the mobile phase was 0.3% acetate (70%) and methanol (30%) at a flow rate of 0.8 mL/min, with separation carried out using an Agilent ZORBAX SB-Aq-C18 column (5 μm, 4.6 × 250 mm) at 50 °C [27].

Total RNA was extracted using Trizol reagent according to the manufacturer’s protocol (Takara Bio Inc., Otsu, Japan). The integrity and purity of the RNA were determined using Trizol™ (Invitrogen, Carlsbad, CA, USA) and electrophoresis using 1% agarose gel. Then the samples (*C. beijerinckii* NCIMB 8052 and *C. beijerinckii* 3304::int) were send to Beijing Genomics Institute (BGI) (Shenzhen, China) for transcriptome assembly and differentially expressed genes (DEGs) analysis (http://www.genomics.cn/index) using Gene Ontology (GO) functional annotation (http://geneontology.org/) and Kyoto Encyclopedia of Genes and Genomes (KEGG) pathway enrichment analysis (http://www.genome.jp/kegg/).

## Additional files

**Additional file 1: Figure S1.** The sequence of Cbei_3304.

**Additional file 2: Table S1.** The differentially expressed genes involved in membrane transport proteins.

**Additional file 3: Table S2.** The differentially expressed genes involved in acetate formation.

**Additional file 4: Table S3.** The differentially expressed genes involved in butyrate formation.

**Additional file 5: Table S4.** The differentially expressed genes involved in butyrate formation.

## Abbreviations

ABC: ATP-binding cassette
MFS: major facilitator superfamily
SAHHC: hemicellulosic hydrolysate of corn cob treated with dilute sulfuric acid
SAHHB: hemicellulosic hydrolysate of bagasse fiber treated with dilute sulfuric acid
LDMICs: lignocellulose-derived microbial inhibitory compounds
ABE: acetone–butanol–ethanol
DCW: dry cell weight
DEGs: differentially expressed genes
60P2-SAHHC: the raw SAHHC containing 60 g/L of reducing sugar
40P2-SAHHC: the raw SAHHC was diluted to contain 40 g/L of reducing sugar
30P2-SAHHC: the raw SAHHC was diluted to contain 30 g/L of reducing sugar
46P2-SAHHB: the raw SAHHB containing 60 g/L of reducing sugar
40P2-SAHHB: the raw SAHHB was diluted to contain 40 g/L of reducing sugar
30P2-SAHHB: the raw SAHHB was diluted to contain 30 g/L of reducing sugar
TPC: total phenolic compounds concentration
