# Supplementary material for: Enhanced phenolic compounds tolerance response of Clostridium beijerinckii NCIMB 8052 by inactivation of Cbei_3304
Source: Microb Cell Fact. 2018 Mar 3;17:35. doi: 10.1186/s12934-018-0884-0 (PMC5834869; doi:10.1186/s12934-018-0884-0)
Supplement: Supplementary file 1 — Additional file 1: Figure S1. The sequence of Cbei_3304. [file 12934_2018_884_MOESM1_ESM.pdf]

The sequence of gene Cbei_3304

http://www.kegg.jp/dbget-bin/www_bget?-f+-n+n+cbe:Cbei_3304

1 ATGACAAAGGTTAATAAATTACCTACAGCAAAACTGTGGAATCCGAAATCATTTATTATA
61 TTTTCCGTATTTTTCTCATTTCTGCCAGCTGGAATCATGTGTGCTTTGAATTATGGACGA
121 TCTGGAAGTCAAAAAAAGAAGTGGATATTTCTTTTAGCAAGCATCTTAGTGTTTATAGCA
181 CTTATAGCACTTCTGCCTATATTATCAATTAATACTTCTATCATATTTTTCAGTATTAAT
241 ATAGCTTTAGGCATAATTCTTATGTTTACACAGTTAAAGCTGTATAATGAACATATTCAA
301 AATGGCGGGCAAAGTGCTTCGTATTTATTTCCTATAATCATAGGGCTTTTGATTTTTTCA
361 TTGTCAGCTGCTTCAATTCTATACTCAATTTACGTGCCAAAAAATGCTCTTGATTATGGC
421 GAAAATCACTTATTTTATACTAATAAGATAACAGAATCCCAAGCGAAAAAACTTGGAGAT
481 TATTTAAATTCAGAAGGATACTTCACTCCTAGTTCAAAAGTGGACGTTAAGATTGATAAA
541 CAAGATACACTCTATATTTTATCTCTCGTTGTAGAAGGTGACTATAAAAGTGATACAAGC
601 TATGTAGAACCTATGAAAGCAATATCAAAAGAGATTTCAAAAAATGTTTTTGAAAATAAT
661 AAAGTTAGAATAGATTTATGCAATGATAGATTCAAGGTTCTTAATTCCATCAATGTTGAT
721 TAA

The sequence of hypothetical protein Cbei_3304

http://www.kegg.jp/dbget-bin/www_bget?-f+-n+a+cbe:Cbei_3304

1 MTKVNKLPTAKLWNPKSFIIFSVFFSFLPAGIMCALNYGRSGSQKKKWIFLLASILVFIA
61 LIALLPILSINTSIIFFSINIALGIILMFTQLKLYNEHIQNGGQSASYLFPIIIGLLIFS
121 LSAASILYSIYVPKNALDYGENHLFYTNKITESQAKKLGDYLNSEGYFTPSSKVDVKIDK
181 QDTLYILSLVVEGDYKSDTSYVEPMKAISKEISKNVFENNKVRIDLCNDRFKVLNSINVD
241
