# Supplementary material for: Enhanced phenolic compounds tolerance response of Clostridium beijerinckii NCIMB 8052 by inactivation of Cbei_3304
Source: Microb Cell Fact. 2018 Mar 3;17:35. doi: 10.1186/s12934-018-0884-0 (PMC5834869; doi:10.1186/s12934-018-0884-0)
Supplement: Supplementary file 2 — Additional file 2: Table S1. The differentially expressed genes involved in membrane transport proteins. [file 12934_2018_884_MOESM2_ESM.pdf]

Table S1 The differentially expressed genes involved in membrane transport proteins

Gene ID	8052-A-RPKM	3304-A-RPKM	8052-S-RPKM	3304-S-RPKM	Description	log2 Ratio-A	log2 Ratio-S
Cbei_3693	107.5720145	178.634639	262.5605268	497.8431653	cobalt ABC transporter ATPase	0.731709059	0.923041286
Cbei_1128	584.052804	186.7619118	379.7394827	647.9624755	phosphate ABC transporter permease	1.644898546	0.770900259
Cbei_1129	583.2018463	84.98978834	139.2678162	280.3220233	phosphate ABC transporter permease	2.778633872	1.009223193
Cbei_1130	521.6177796	52.52530366	95.09331675	153.5370442	phosphate ABC transporter ATPase	-3.311908543	0.691170924
Cbei_1081	563.4710243	2596.739276	1649.257993	735.9334627	ABC transporter	2.20428784	-1.164169856
Cbei_1765	43.44484994	82.46624105	32.45952327	77.89525483	ABC transporter	0.924618482	1.262893634
Cbei_1766	47.1890959	96.33946169	42.11641204	111.3313246	ABC transporter	1.029673332	1.40240513
Cbei_2089	4.618011852	6.91010512	7.470381267	13.86325631	ABC transporter	0.581435782	0.892012388
Cbei_2145	6.907554893	23.98924557	11.65415009	17.16871702	ABC transporter	1.796140761	0.558938439
Cbei_5045	80.60529778	1111.600395	296.7544559	211.5041669	ABC transporter	3.785619779	-0.488583606
Cbei_5046	69.87062849	663.5872339	223.0920023	179.0258709	ABC transporter	3.247528109	-0.317470709
Cbei_3331	170.9779847	907.9123178	496.0243562	773.3929734	ABC transporter	2.408742401	0.640790694
Cbei_4190	28.76898526	131.4761669	66.13119642	70.8536945	sulfate ABC transporter ATPase	2.192215061	0.099512078
Cbei_4191	22.93914384	146.5817774	52.61087984	63.97207938	sulfate ABC transporter ATPase	2.675822311	0.282081201
Cbei_4192	30.9285513	107.252563	62.7081133	81.06615602	sulfate ABC transporter permease	1.794000961	0.370447621
Cbei_4193	24.1267558	78.45193128	46.43096929	66.50156454	sulfate ABC transporter substrate-binding protein	1.701175026	0.518300884
Cbei_1127	307.691339	89.59453889	180.2073263	299.303124	phosphate binding protein	-1.780001132	0.731949672
Cbei_1762	42.37860177	127.5055822	16.65373286	12.06843228	extracellular ligand-binding receptor	1.589152516	-0.464607308
Cbei_1767	43.24955888	68.38866923	35.86207915	121.9289419	extracellular ligand-binding receptor	0.661071898	1.765509577
Cbei_1763	50.40434642	135.1717573	47.54205753	159.4603137	inner-membrane translocator	1.423173698	1.745921166
Cbei_1764	45.95626777	99.93677416	30.73094617	113.8129914	inner-membrane translocator	1.120754013	1.888901155
Cbei_5043	13.49555975	163.8037142	49.34615571	42.36230576	inner-membrane translocator	3.601411349	-0.22015658
Cbei_5044	52.62430069	669.1807867	174.3867627	144.7804487	inner-membrane translocator	3.668594962	-0.26842374
Cbei_3299	391.6736954	246.4791246	812.3883629	1182.641182	multidrug ABC transporter ATPase	-0.668186777	0.541770942
Cbei_3300	297.9958129	252.3172374	870.981744	1151.899816	multidrug ABC transporter ATPase	-0.24005329	0.403300862
Cbei_3884	0.496310258	2.313633336	0.747492332	0.694332467	multidrug ABC transporter ATPase	2.220846067	-0.106432146
Cbei_3445	22.71121924	8.266293975	16.33228054	14.81296673	small multidrug resistance protein	-1.458092582	-0.140865643
Cbei_3916	1.22839883	10.34991383	3.042370426	8.678506529	major facilitator superfamily transporter	3.074767807	1.512250973
Cbei_3028	116.7776223	38.72929923	37.39323546	49.5261245	major facilitator superfamily transporter	-1.592266539	0.405412426

A:acidogenesis; S:solventogenesis;

log2 Ratio-A: Comparison of DEGs after Cbei_3304 inactivation in acidogenesis;

log2 Ratio-S: Comparison of DEGs after Cbei_3304 inactivation in solventogenesis.
