# Supplementary material for: Enhanced phenolic compounds tolerance response of Clostridium beijerinckii NCIMB 8052 by inactivation of Cbei_3304
Source: Microb Cell Fact. 2018 Mar 3;17:35. doi: 10.1186/s12934-018-0884-0 (PMC5834869; doi:10.1186/s12934-018-0884-0)
Supplement: Supplementary file 3 — Additional file 3: Table S2. The differentially expressed genes involved in acetate formation. [file 12934_2018_884_MOESM3_ESM.pdf]

Table S2 The differentially expressed genes involved in acetate formation

Gene ID	8052-A-RPKM	3304-A-RPKM	8052-S-RPKM	3304-S-RPKM	Description	log2 Ratio-A	log2 Ratio-S
Cbei_1164	9381.981146	3026.059484	4285.860927	1596.2321	phosphotransacetylase	-1.632452254	-1.424914555
Cbei_1165	8173.274456	2001.011618	4379.862246	1489.8676	acetate kinase	-2.030184637	-1.555701395
Cbei_4233	5.413054186	2.243009608	4.710388188	6.4368816	acetate kinase	-1.27100703	0.450515979

A:acidogenesis; S:solventogenesis;

log2 Ratio-A: Comparison of DEGs after Cbei_3304 inactivation in acidogenesis;

log2 Ratio-S: Comparison of DEGs after Cbei_3304 inactivation in solventogenesis.
