# Supplementary material for: Enhanced phenolic compounds tolerance response of Clostridium beijerinckii NCIMB 8052 by inactivation of Cbei_3304
Source: Microb Cell Fact. 2018 Mar 3;17:35. doi: 10.1186/s12934-018-0884-0 (PMC5834869; doi:10.1186/s12934-018-0884-0)
Supplement: Supplementary file 4 — Additional file 4: Table S3. The differentially expressed genes involved in butyrate formation. [file 12934_2018_884_MOESM4_ESM.pdf]

Table S3 The differentially expressed genes involved in butyrate formation

Gene ID	8052-A-RPKM	3304-A-RPKM	8052-S-RPKM	3304-S-RPKM	Description	log2 Ratio-A	log2 Ratio-S
Cbei_0203	4654.386951	2984.239153	4877.568104	1854.496712	phosphate butyryltransferase	-0.641228001	-1.395134307
Cbei_0204	5607.200368	3150.004566	4009.982574	1598.150501	butyrate kinase	-0.831926704	-1.327192691
Cbei_4006	8.646734921	10.76791214	7.758286428	8.782965354	butyrate kinase	0.316511176	0.178970073

A:acidogenesis; S:solventogenesis;

log2 Ratio-A: Comparison of DEGs after Cbei_3304 inactivation in acidogenesis;

log2 Ratio-S: Comparison of DEGs after Cbei_3304 inactivation in solventogenesis.
