# Supplementary material for: Enhanced phenolic compounds tolerance response of Clostridium beijerinckii NCIMB 8052 by inactivation of Cbei_3304
Source: Microb Cell Fact. 2018 Mar 3;17:35. doi: 10.1186/s12934-018-0884-0 (PMC5834869; doi:10.1186/s12934-018-0884-0)
Supplement: Supplementary file 5 — Additional file 5: Table S4. The differentially expressed genes involved in butyrate formation. [file 12934_2018_884_MOESM5_ESM.pdf]

Table S4 The differentially expressed genes involved in butanol formation

Gene ID	8052-A-RPKM	3304-A-RPKM	8052-S-RPKM	3304-S-RPKM	Description	log2 Ratio-A	log2 Ratio-S
Cbei_0411	3907.03077	6495.63912	2150.59109	2092.123953	acetyl-CoA acetyltransferase	0.733398865	-0.039764909
Cbei_3630	75.45494514	47.4361336	123.5847649	167.634472	acetyl-CoA acetyltransferase	-0.669629029	0.439817949
Cbei_2653	6.778732994	2.4968058	4.688780903	8.851146454	3-ketoacid-CoA transferase	-1.440932031	0.916651467
Cbei_3820	6.027447256	4.73681987	5.947610402	2.507891519	3-hydroxyacyl-CoA dehydrogenase, NAD-binding	-0.347628311	-1.245835202
Cbei_4279	70.15646288	63.656726	42.46486321	35.76140963	enoyl-CoA hydratase	-0.140263055	-0.247865997
Cbei_2038	20.11978873	40.4873765	26.93545922	23.77966459	enoyl-CoA hydratase	1.008857006	-0.179778295
Cbei_2883	141.5542159	1779.28213	110.8924546	52.40976775	acyl-CoA dehydrogenase domain protein	3.651868664	-1.081253583
Cbei_2035	5.27155505	9.2209338	10.67274797	7.616644449	acyl-CoA dehydrogenase	0.806684255	-0.486704227
Cbei_4542	7.172771625	5.99808315	6.941624699	7.979341803	acyl-CoA dehydrogenase	-0.258029176	0.200996379
Cbei_0305	5.960408875	6.92178071	7.471274676	9.082109483	acetaldehyde dehydrogenase	0.215731935	0.281673026
Cbei_1932	151.5783726	179.183935	142.2105747	87.14888347	NADH-dependent butanol dehydrogenase	0.241377377	-0.706474661
Cbei_0558	63.12612171	161.722349	69.44771808	56.23688259	aldo/keto reductase	1.357210036	-0.304410668
Cbei_2676	108.133033	97.2453198	85.33633787	122.5206064	aldo/keto reductase	-0.153106589	0.521792307
Cbei_3134	206.5005875	117.665645	120.3504182	114.7190398	aldo/keto reductase	-0.811452728	-0.069136302
Cbei_0764	106.2192867	72.2487764	157.0231685	133.7845226	aldo/keto reductase	-0.556000688	-0.23106622

A:acidogenesis;   S:solventogenesis;

log2 Ratio-A: Comparison of DEGs after Cbei_3304 inactivation in acidogenesis;

log2 Ratio-S: Comparison of DEGs after Cbei_3304 inactivation in solventogenesis.
